# Long-term natural history of thyroid peroxidase antibodies in a population-based cohort: Findings from 18 years of follow-up in Tehran Thyroid Study (TTS)

**DOI:** 10.1016/j.jtauto.2026.100358

**Published:** 2026-02-16

**Authors:** Mohamadamin Tarighat-Payma, Ladan Mehran, Safdar Masoumi, Maryam Tohidi, Atieh Amouzegar, Fereidoun Azizi, Elizabeth N. Pearce

**Affiliations:** a Endocrine Research Center, Research Institute for Endocrine Disorders, Research Institute for Endocrine Sciences, Shahid Beheshti University of Medical Sciences, Tehran, Iran; b Prevention of Metabolic Disorders Research Center, Research Institute for Metabolic and Obesity Disorders, Research Institute for Endocrine Sciences, Shahid Beheshti University of Medical Sciences, Tehran, Iran; c Section of Endocrinology, Diabetes, Nutrition and Weight Management, Boston University Chobanian & Avedisian School of Medicine, Boston, MA, USA

**Keywords:** Thyroid peroxidase antibody, Autoimmune thyroid disease, Trend, Trajectory, Longitudinal study

## Abstract

**Background:**

Thyroid peroxidase antibody (TPOAb) is considered a highly sensitive marker of autoimmune thyroid diseases (AITD). Limited longitudinal data exist regarding its long-term natural history in the general population. This study aimed to assess risk factors for TPOAb positivity and its 18-year prevalence, incidence, trajectories, and projected prevalence for the year 2030 in a population-based cohort.

**Methods:**

A total of 5,438 adults were recruited at first visit (1999-2002) and followed across four subsequent visits up to 2018 in the iodine-sufficient population-based Tehran Thyroid Study (TTS). The age- and sex-standardized prevalence of TPOAb positivity was calculated, and the projected prevalence in 2030 was estimated using Poisson Generalized Linear Mixed Model (Poisson GLMM). Longitudinal trajectories of TPOAb were identified using latent class growth mixture model (LCGMM). Cox proportional hazards models were used to examine associations between potential risk factors and TPOAb positivity.

**Results:**

The prevalence increased progressively from 11.7% at the first visit (1999-2002) to 16.3% at the fifth visit (2015-2018), and is projected to reach 21.04% in 2030. Four distinct TPOAb trajectories were identified: Low-stable (81.4%), Low-increasing (2.9%), High-decreasing (2.4%), and High-stable (13.3%). The overall incidence rate of TPOAb positivity was 5.6 per 1,000 person-years, higher among women and individuals aged <40 years. In multivariable analysis, female sex (Hazard Ratio (HR) = 1.61; 95% CI: 1.15-2.27) and elevated TSH ≥5 mU/L (HR = 2.69; 95% CI: 1.57-4.63) were significant positive predictors of TPOAb positivity, while age between 40 and 60 years was inversely associated with incident TPOAb positivity (HR = 0.71; 95% CI: 0.55-0.90).

**Conclusions:**

This is the first and longest study worldwide that demonstrated a persistent rise in TPOAb positivity across five repeated measurements in an iodine-sufficient population, driven by female sex, age <40, and TSH ≥5 mU/L, which is projected to reach 21.04% in 2030. Trajectory patterns of TPOAb showed that the majority of participants had consistently low-stable levels of TPOAb.

## 1 Introduction

Thyroid peroxidase (TPO) is a transmembrane enzyme located on the apical surface of thyroid follicular cells, and it plays an essential role in thyroid hormone synthesis [1]. Loss of immune tolerance to TPO leads to the development of thyroid peroxidase antibody (TPOAb), which is present in 90-95% of Hashimoto's thyroiditis and 75% of those with Graves' disease [1,2]. Accordingly, TPOAb is considered a highly sensitive serological marker for autoimmune thyroid diseases (AITD). Beyond thyroid dysfunction, AITD is associated with several comorbidities [2], including non-thyroidal autoimmune diseases [3], cardiovascular diseases [4,5], malignancies [4,5], adverse neurodevelopmental outcomes in offspring [6,7], and a higher risk of all-cause and cause-specific mortality [2,4,5].

The prevalence of TPOAb positivity has been reported in several large studies, ranging from 2.8% to 18.5% worldwide [[8], [9], [10], [11], [12]]. A few longitudinal studies have investigated the incidence of TPOAb positivity, ranging from about 3.5 to 8.5 per 1,000 person-years [[12], [13], [14], [15]]. In Iran, we previously reported the incidence of TPOAb positivity to be 7.12 per 1.000 person-years during the period from 2000 to 2012 [12]. Female sex, age, genetic factors, family history of AITD, smoking status, iodine status, and alcohol consumption have been found to be the risk factors for TPOAb development [4,5,12,[16], [17], [18], [19]].

Although several studies have investigated the prevalence and risk factors of TPOAb positivity, few longitudinal studies have examined the long-term incidence of TPOAb positivity. Most of these studies lack repeated measurements, and none of them have assessed the trend of TPOAb based on follow-up measurements over an extended period. Therefore, we aimed to investigate risk factors for TPOAb positivity and its 18-year prevalence, incidence, trajectories, and projected prevalence for the year 2030.

## 2 Methods

### 2.1 Study design and participants

The Tehran Thyroid Study (TTS) is a long-term, community-based cohort study within the framework of the Tehran Lipid and Glucose Study (TLGS) [20], investigating the prevalence, incidence, and natural history of thyroid diseases and their long-term outcomes in the iodine-sufficient population [21]. National iodine deficiency disorders (IDD) monitoring surveys conducted every 5 years from 1996 to 2018 in Iran indicated that this population was iodine sufficient throughout the study period [[22], [23], [24]]. A detailed description of the design and methodology of this study has been published previously [25]. Study examinations were conducted at first visit (1999-2002), second visit (2002-2005), third visit (2005-2008), fourth visit (2008-2011), and fifth visit (2015-2018).

**Fig. 1:**
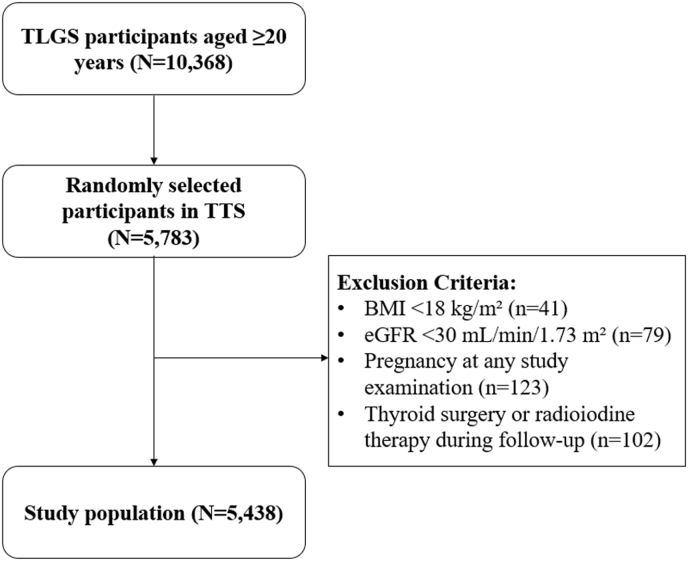
**Flowchart of the study participants. Participants from the TLGS were randomly selected to participate in the TTS.** After excluding individuals based on predefined criteria, the final study population was included for the main analysis. Abbreviations: TLGS, Tehran Lipid and Glucose Study; TTS, Tehran Thyroid Study; Body Mass Index, BMI; eGFR, estimated glomerular filtration rate.

From 10,368 TLGS participants who were aged ≥20 years, 5,783 individuals were randomly selected to participate in the TTS study. Exclusion criteria in this study were body mass index (BMI) < 18 kg/m^2^ (n = 41), estimated glomerular filtration rate (eGFR) < 30 mL/min/1.73 m^2^ (n = 79); pregnancy at any study examination (n = 123); and a history of thyroid surgery or radioiodine therapy reported during follow-up (n = 102). As a result, 5,438 participants were included in the main analysis (Fig. 1).

### 2.2 Procedures

Trained physicians collected data on participants’ basic demographics, medication history, family and personal history of thyroid diseases, radioactive iodine exposure, education level, physical activity, and smoking habits. Physical activity was measured using the Lipid Research Clinic (LRC) and the Modifiable Activity Questionnaire (MAQ), with results expressed on the metabolic equivalent of task (MET) scale. Participants wore light clothing and no shoes during anthropometric measurements. Weight and height were measured using a digital electronic weighing scale (Seca 707; range, 0.1-150 kg; Seca, Hanover, MD), with an accuracy of up to 100 g, and a stadiometer tape meter, respectively. In order to calculate BMI, weight (kg) was divided by height (meters) squared. The waist circumference was measured in centimeters at the narrowest waist level.

Blood samples were taken between 7:00 a.m. and 9:00 a.m. from all study participants, following an overnight fast of 12-14 h. Free thyroxine (FT4) and thyroid-stimulating hormone (TSH) were determined on −70 °C stored serum samples by the electro-chemiluminescence immunoassay (ECLIA) method, using Roche Diagnostics kits & Roche/Hitachi Cobas e−411 analyzer (GmbH, Mannheim, Germany). Lyophilized quality control material (Lyphochek Immunoassay plus Control, Bio-Rad Laboratories) was used to monitor the accuracy of the assay; intra- and inter-assay coefficients of variation (CVs) were 1.3% and 3.7% for FT4 and 1.5% and 4.5% for TSH determinations, respectively. TPOAb was measured by immune-enzymometric assay (IEMA) using commercial kits (Monobind, Costa Mesa, CA, USA) and the Sunrise ELISA reader (Tecan Co., Salzburg, Austria); intra- and inter-assay CVs were 3.9% and 4.7%, respectively. All laboratory measurements were performed in the same laboratory by skilled laboratory technicians.

This study conformed to the ethical guidelines of the Helsinki Declaration and was approved by the ethics committee of Research Institute for Endocrine Sciences (RIES) of Shahid Beheshti University of Medical Sciences (code: IR.SBMU.ENDOCRINE.REC.1404.030). All participants provided informed written consents.

### 2.3 Definitions

TPOAb positivity was defined as a TPOAb >35 IU/mL in women and TPOAb >32.8 IU/mL in men [26]. The TSH reference range was defined as 0.32-5.06 mU/L [27]. Participants who tested positive for TPOAb in at least 4 out of 5 study measurements were classified as the persistent group. Smokers were defined as individuals who used tobacco either occasionally or daily. Non-smokers were defined as those who had never smoked or were past smokers. BMI was categorized into three groups: normal weight (18.5-24.9 kg/m^2^), overweight (25-29.9 kg/m^2^), and obese (≥30 kg/m^2^). Education levels were categorized based on the number of years and degree attained: primary school (<6 years), high school (6-12 years), and higher education (>12 years). Participants engaging in less than 600 MET minutes per week were classified as having low physical activity.

### 2.4 Statistical analysis

The baseline characteristics of the participants were reported as mean [Standard Deviation (SD)] or median [Interquartile Range (IQR)] for continuous variables and as count (percentage) for categorical variables. Comparisons of baseline characteristics between participants were performed using Student's t-test and one-way ANOVA for continuous variables and the Chi-square test for categorical variables. The age- and sex-standardized prevalence of TPOAb was reported as percentages with 95% confidence intervals and was predicted in 2030 using the Poisson Generalized Linear Mixed Model (Poisson GLMM). The incidence rate of TPOAb positivity was calculated as the number of new cases during the follow-up period, divided by the person-time at risk throughout the observation period. Given the lack of precise onset timing for TPOAb positivity, the event time was estimated as the half-time survival between the pre-follow-up measurement and the first follow-up measurement. Univariate Cox regression analysis was performed, and variables with a P < 0.2 in the univariate analysis were selected for inclusion in the multivariate model. We analyzed TPOAb trajectories using latent latent class growth mixture model (LCGMM) within the “lcmm” package in R. Model fit was evaluated using the Bayesian Information Criterion (BIC), posterior probability (>0.7), average posterior probability of group membership, and the root mean square error of approximation (RMSEA). The final model identified four distinct trajectories: a low-stable class, a high-stable class, a low-increasing class, and a high-decreasing class. The statistical analyses were performed with STATA 14 (StataCorp, College Station, TX, USA) and R 3.0.3 (R Foundation for Statistical Computing, Vienna, Austria). A two-sided P < 0.05 was considered statistically significant.

## 3 Results

A total of 5,438 participants (57.6% women) with a mean age of 40.5 ± 14.6 years were included in this study. Median serum TSH levels were higher in women (1.74 mU/L; IQR: 1.0-2.5) than in men (1.29 mU/L; IQR: 0.79-1.9) (P < 0 0.001). Mean FT4 levels were lower in women (1.2 ± 0.3 ng/dL) compared to men (1.3 ± 0.7 ng/dL) (P < 0.001). The median TPOAb level in the overall population was 5.86 IU/mL (IQR: 3.3-12.2). Women had significantly higher TPOAb levels (6.44 IU/mL; IQR: 3.5-15.9) than men (5.19 IU/mL; IQR: 3.1-9.6) (P < 0.001) (Table 1). Additionally, the baseline characteristics of participants across the identified four trajectory classes are summarized in Supplementary Table 1.

**Table 1:** Baseline characteristics of the study population.

	Total (n = 5,438)	Men (n = 2,305)	Women (n = 3,133)	P value
Age (years)	40.5 ± 14.6	41.4 ± 15.3	40.0 ± 14.1	<0.001
BMI (kg/m2)	26.7 ± 4.5	25.7 ± 3.9	27.3 ± 4.8	<0.001
Waist circumference (cm)	87.1 ± 11.9	87.8 ± 11.1	86.6 ± 12.3	<0.001
Education level				<0.001
Primary school	2121 (39.0)	724 (31.4)	1397 (44.6)	
High school	2561 (47.1)	1148 (49.8)	1413 (45.1)	
Higher education	756 (13.9)	433 (18.8)	323 (10.3)	
Smoking status				<0.001
Non-smoker	4818 (88.6)	1763 (76.5)	3055 (97.5)	
Smoker	620 (11.4)	542 (23.5)	78 (2.5)	
Low physical activity	2034 (37.4)	997 (43.2)	1037 (33.1)	<0.001
TSH (mU/L)	1.51 (0.8-2.5)	1.29 (0.79-1.9)	1.74 (1.0-2.5)	<0.001
FT4 (ng/dl)	1.2 ± 0.5	1.3 ± 0.7	1.2 ± 0.3	<0.001
TPOAb (IU/mL)	5.86 (3.3-12.2)	5.19 (3.1-9.6)	6.44 (3.5-15.9)	<0.001

Categorical variables were reported as count (percentage), and continuous variables as mean ± SD or median (IQR). Abbreviations: BMI, body mass index; TSH, thyroid-stimulating hormone; FT4, free T4; TPOAb, thyroid peroxidase antibody.

**Fig. 2:**
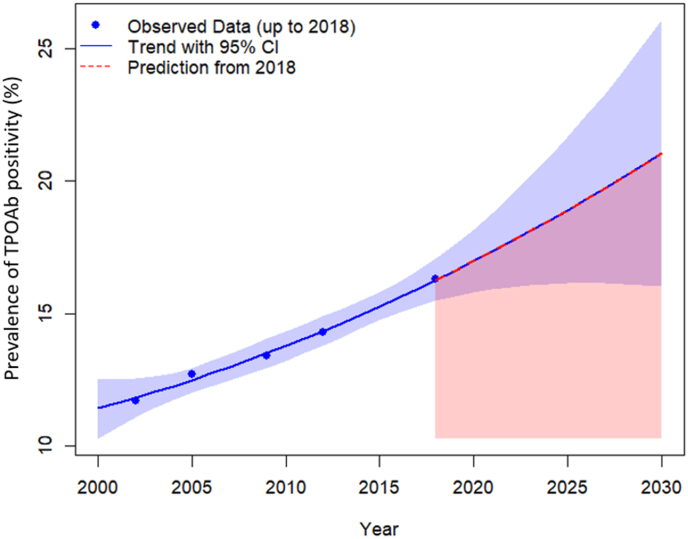
**Age- and sex-standardized prevalence of TPOAb positivity over 18 years of follow-up, with projection to the year 2030.** Blue points represent observed prevalence measures (data up to 2018). The solid blue line represents the fitted trend, and the blue shaded band indicates the 95% confidence interval (CI) around that trend. The shaded panel from 2019 to 2030 indicates the projection window; the dashed red line represents the model-based projection from 2018. Estimates are standardized to the study population by age and sex. Abbreviations: TPOAb, thyroid peroxidase antibody; CI, confidence interval.

Table 2 shows the age- and sex-standardized prevalence of TPOAb positivity in five repeated measurements from 1999 to 2018 based on sex, age, smoking status, and BMI categories. The prevalence of TPOAb positivity increased progressively over time, recorded at 11.7% (95% CI: 9.5-14.3) at the first visit, 12.7% (95% CI: 11.2-14.2) at the second visit, 13.4% (95% CI: 11.8-15.2) at the third visit, 14.3% (95% CI: 12.7-16.0) at the fourth visit, and 16.3% (95% CI: 14.6-18.3) at the fifth visit. The prevalence of TPOAb positivity in the cohort is projected to reach 21.04% (95% CI: 16.01-26.08) in 2030 (Fig. 2) (Supplementary Table 2). Among women, the prevalence of TPOAb positivity increased from 15.3% (95% CI: 12.3-18.9) to 19.6% (95% CI: 17.2-22.2), while in men the prevalence of TPOAb positivity increased from 5.7% (95% CI: 3.5-9.1) to 11.2% (95% CI: 8.9-13.9) (P < 0.001). These uprising trends were observed in individuals under 60 years old, smokers and non-smokers, and normal, overweight, and obese individuals (Table 2). Out of all participants, 220 (4.05%) tested positive in all five visits, 4,330 participants (79.62%) tested negative for TPOAb across all visits, and 888 participants (16.33%) tested positive in at least one to at most four visits (Supplementary Table 3).

**Table 2:** Age- and sex-standardized prevalence of TPOAb positivity based on sex, age, smoking status, and BMI categories over 18 years of follow-up.

	1999-2002 (First visit)	2002-2005 (Second visit)	2005-2008 (Third visit)	2008-2011 (Fourth visit)	2015-2018 (Fifth visit)
	Prevalence rate (95 % CI)	Prevalence rate (95 % CI)	Prevalence rate (95 % CI)	Prevalence rate (95 % CI)	Prevalence rate (95 % CI)
Total	11.7 (9.5-14.3)	12.7 (11.2-14.2)	13.4 (11.8-15.2)	14.3 (12.7-16.0)	16.3 (14.6-18.3)
Sex					
Men	5.7 (3.5-9.1)	8.1 (6.2-10.5)	8.4 (6.4-10.9)	9.0 (7.2-11.4)	11.2 (8.9-13.9)
Women ∗	15.3 (12.3-18.9)	15.7 (13.6-18.2)	16.6 (14.3-19.1)	17.7 (15.6-20.2)	19.6 (17.2-22.2)
Age					
< 40 years	10.8 (7.7-15.0)	11.5 (9.2-14.2)	12.4 (10.0-15.2)	13.5 (11.2-16.2)	15.7 (13.1-18.7)
40 – 60 years	12.6 (11.1-14.3)	14.8 (13.3-16.6)	15.2 (13.7-16.9)	16.0 (14.5-17.6)	18.8 (17.1-20.6)
≥ 60 years	15.0 (13.1-17.1)	15.1 (13.2-17.1)	15.5 (13.7-17.6)	14.9 (13.2-16.8)	14.4 (12.7-16.4)
Smoking status					
Non-smoker	11.9 (9.7-14.5)	12.9 (11.3-14.8)	13.7 (11.9-15.8)	14.5 (12.8-16.5)	16.5 (14.6-18.5)
Smoker	8.7 (2.9-23.6)	10.8 (6.0-18.8)	13.3 (7.2-23.3)	11.7 (6.7-19.4)	15.3 (10.6-21.6)
BMI					
Normal weight	13.4 (9.0-19.5)	11.8 (9.1-15.3)	14.0 (11.0-17.7)	15.5 (12.5-19.1)	16.3 (13.1-20.2)
Overweight	10.3 (7.6-13.9)	12.4 (10.1-15.1)	12.7 (10.3-15.6)	13.2 (10.9-15.9)	16.0 (13.4-19.0)
Obese	11.9 (7.9-17.5)	13.7 (10.8-17.1)	13.7 (10.7-17.3)	14.2 (11.4-17.5)	16.1 (13.0-19.7)

Reported values are age- and sex-standardized prevalence of TPOAb positivity, expressed as percentage with 95% confidence interval (CI). ∗Significant difference in trend of TPOAb positivity between men and women (P < 0.001). Abbreviations: BMI, body mass index; TPOAb, thyroid peroxidase antibody.

**Fig. 3:**
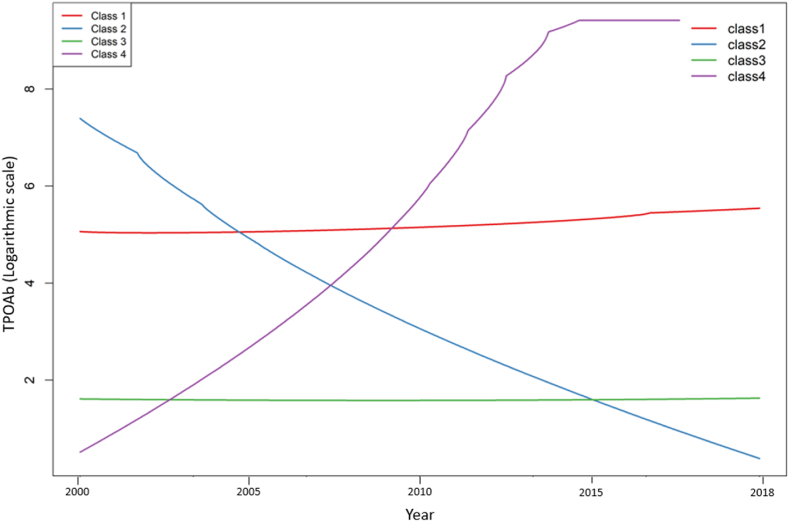
**Class-specific longitudinal trajectories of TPOAb over 18 years of follow-up.** Four latent trajectory classes were identified: Class 1 (13.3%): high-stable, gradual increase from 5.1 to 5.7 (logarithmic scale); Class 2 (2.4%): high-decreasing, decline from 7.8 to 0.8 (logarithmic scale); Class 3 (81.4%): low-stable, stable low levels of 1.8 to 1.9 (logarithmic scale); Class 4 (2.9%): low-increasing, increase from 0.9 to 9.7 (logarithmic scale). Colored lines show model-estimated mean trajectories. TPOAb values are plotted on the logarithmic scale. Abbreviations: TPOAb, thyroid peroxidase antibody.

**Fig. 4:**
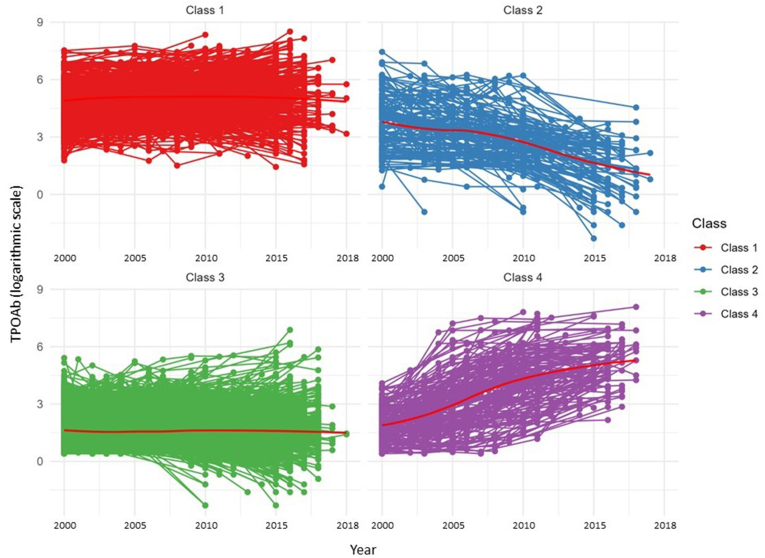
**Longitudinal variations of TPOAb over 18 years of follow-up, stratified by identified classes.** Each participant's trajectories are shown in class colors. Class-specific mean trajectories are plotted as red lines. Classes: Class 1 (high-stable, 13.3%), Class 2 (high-decreasing, 2.4%), Class 3 (low-stable, 81.4%), Class 4 (low-increasing, 2.9%). TPOAb values are plotted on the logarithmic scale. Abbreviations: TPOAb, thyroid peroxidase antibody.

LCGMM identified four distinct trajectories of TPOAb levels over 18 years of follow-up. Class 1 (High-stable class), comprising 13.3% of participants, showed a gradual increase in TPOAb levels. Class 2 (High-decreasing class), comprising 2.4% of participants, showed a marked decrease in TPOAb levels. Class 3 (Low-stable class), comprising 81.4% of participants, demonstrated stable TPOAb levels. Class 4 (Low-increasing class), comprising 2.9% of participants, showed a notable rise in TPOAb levels (Fig. 3) (Fig. 4) (Supplementary Table 4). In addition, longitudinal trends of TSH, FT4, and BMI across study phases for each identified trajectory class are presented in Supplementary Table 5 and Supplementary Fig. 1.

The overall incidence of TPOAb positivity in the total population was 5.6 (95% CI: 5.1-6.3) per 1,000 person-years (cumulative 18-year incidence of 10.2%), which was significantly higher in women (7.3, 95% CI: 6.5-8.3) than compared to men (3.6, 95% CI: 2.9-4.3). The highest incidence of TPOAb positivity was in those <40 years of age (6.6; 95% CI: 5.8-7.5). Participants classified as non-smokers had a higher overall incidence of TPOAb positivity, at 5.6 (95% CI: 5.3-6.6), compared to 3.8 (95% CI: 2.6-5.5) in smokers. Across BMI categories, normal weight individuals showed the highest incidence of TPOAb positivity at 6.1 (95% CI: 5.2-7.2). The incidence of TPOAb positivity was highest in participants with TSH ≥5 mU/L (17.1; 95% CI: 12.5-23.4). In all these subgroups, the incidence was consistently higher in women than in men (Table 3).

**Table 3:** Incidence rate (IR, per 1000 person-years) of TPOAb positivity based on sex, age, smoking status, BMI categories, and TSH categories over 18 years of follow-up.

	Total		Men		Women	
	Events	IR (95% CI)	Events	IR (95% CI)	Events	IR (95% CI)
Total	356	5.6 (5.1-6.3)	100	3.6 (2.9-4.3)	256	7.3 (6.5-8.3)∗
Age						
< 40 years	229	6.6 (5.8-7.5)	66	4.3 (3.4-5.5)	163	8.5 (7.3-9.9)
40 – 60 years	101	4.6 (3.8-5.6)	23	2.6 (1.7-3.9)	78	6.0 (4.8-7.5)
≥ 60 years	26	3.9 (2.6-5.7)	11	2.8 (1.6-5.1)	15	5.3 (3.2-8.8)
Smoking status						
Non-smoker	328	5.6 (5.3-6.6)	77	3.6 (2.8-4.4)	251	7.3 (6.5-8.3)
Smoker	27	3.8 (2.6-5.5)	23	3.6 (2.4-5.4)	4	4.9 (1.8-13.0)
BMI						
Normal weight	147	6.1 (5.2-7.2)	53	4.4 (3.4-5.7)	94	7.8 (6.4-9.6)
Overweight	131	5.2 (4.4-6.2)	30	2.5 (1.8-3.6)	101	7.7 (6.3-9.3)
Obese	74	5.5 (4.4-7.0)	17	4.3 (2.7-6.9)	57	6.1 (4.7-7.9)
TSH (mU/L)						
< 0.3	20	5.6 (3.6-8.7)	5	3.9 (1.6-9.3)	15	6.6 (4.0-10.9)
0.3 -2	142	3.6 (3.0-4.2)	56	2.8 (2.1-3.6)	86	4.4 (3.6-5.5)
2 – 5	154	8.8 (7.5-10.3)	36	5.7 (4.1-7.9)	118	10.5 (8.8-12.6)
≥ 5	39	17.1 (12.5-23.4)	3	7.6 (2.5-23.7)	36	19.0 (13.7-26.4)

Reported values are number of incident cases (Events) and incidence rate (IR) per 1000 person-years with 95% confidence interval (CI). ∗Significant difference in trend of TPOAb positivity between men and women (P < 0.001). IRs are age- and sex-standardized unless stratified. BMI, body mass index; TSH, thyroid stimulating hormone; TPOAb, thyroid peroxidase antibody.

In the multivariate analysis, female sex was associated with a higher hazard of developing TPOAb positivity (HR = 1.61, 95 % CI: 1.15-2.27; P = 0.006). Similarly, TSH ≥5 mU/L was associated with a greater hazard of TPOAb positivity (HR = 2.69, 95% CI 1.57-4.63; P < 0.001). On the other hand, age 40-60 years was associated with a lower risk of incident TPOAb positivity compared to other age groups (HR = 0.71, 95% CI 0.55-0.90; P = 0.006). No significant associations were observed for smoking status, BMI, and parity in the multivariate analysis (Table 4).

**Table 4:** Cox proportional hazards regression analysis of TPOAb based on sex, age, smoking status, BMI categories, TSH categories, and parities over 18 years of follow-up.

	Univariate		Multivariate	
	HR (95 % CI)	P value	HR (95 % CI)	P value
Sex				
Male	1.0 (Reference)	-	1.0 (Reference)	-
Female	2.06 (1.63-2.59)	<0.001	1.61 (1.15-2.27)	0.006
Age				
< 40 yr.	1.0 (Reference)	-	1.0 (Reference)	-
40-60 yr.	0.68 (0.55-0.85)	0.001	0.71 (0.55-0.90)	0.006
≥ 60 yr.	0.56 (0.37-0.84)	0.008	0.67 (0.44-1.02)	0.060
Smoking status				
Non-smoker	1.0 (Reference)	-	1.0 (Reference)	-
Smoker	0.63 (0.43-0.94)	0.022	1.0 (0.66-1.50)	0.99
BMI				
Normal weight	1.0 (Reference)	-	-	-
Overweight	0.86 (0.68-1.09)	0.213	-	-
Obese	0.91 (0.68-1.20)	0.484	-	-
TSH (mU/L)				
< 0.3	1.0 (Reference)	-	1.0 (Reference)	-
0.3 - 2	0.64 (0.40-1.01)	0.058	0.66 (0.41-1.06)	0.090
2 – 5	1.57 (0.99-2.51)	0.057	1.49 (0.94-2.38)	0.090
≥ 5	3.02 (1.76-5.18)	<0.001	2.69 (1.57-4.63)	<0.001
Number of parities				
0	1.0 (Reference)	-	1.0 (Reference)	-
≥ 1	1.61 (1.30-1.98)	0.001	1.08 (0.8-1.46)	0.600

Hazard ratios (HRs) with 95% confidence intervals (CIs) were estimated using Cox proportional hazards regression. Abbreviations: BMI, body mass index; TSH, thyroid stimulating hormone; TPOAb, thyroid peroxidase antibody.

## 4 Discussion

This study is the longest-duration cohort to date that evaluates the natural history and trend of TPOAb positivity over 18 years across five repeated measurements, and its projection for 2030 in an iodine-sufficient country. The overall prevalence of TPOAb positivity rose from 11.7% at the first visit (1999-2002) to 16.3% at the fifth visit (2015-2018), and is projected to reach 21.04% in 2030. Similar increasing trends were observed across subgroups defined by sex, age <60, smokers and non-smokers, and normal, overweight, and obese individuals. The majority of participants exhibited consistently low-stable levels, while smaller subgroups showed low-increasing, high-decreasing, or high-stable trends over time. The incidence of TPOAb positivity was 5.6 per 1,000 person-years (cumulative 18-year incidence of 10.2%) and was significantly higher in women at 7.3 per 1.000 person-years (cumulative incidence of 13.1%). Additionally, we found that female sex, TSH ≥5 mU/L, and age <40 years were risk factors for TPOAb positivity.

The trend of TPOAb over 18 years showed four distinct trajectories. The dominant low-stable group (Class 3, 81.4%) showed low and stable TPOAb levels, the high-stable group (Class 1, 13.3%) demonstrated stable elevated TPOAb levels, the low-increasing group (Class 4, 2.9%) showed a notable rise in TPOAb levels, and the high-decreasing group (Class 2, 2.4%) showed a marked decline in TPOAb levels. To date, no study has comprehensively assessed the long-term trend of TPOAb positivity across repeated measurements. While some studies have reported decreasing prevalence of Hashimoto's thyroiditis [28,29] or stable prevalence of AITD [30], these findings do not specifically reflect the trend of TPOAb positivity over time. Such variations may relate to changes in diagnostic criteria and the persistence of TPOAb despite improvements of symptoms with thyroid hormone therapy [28]. In the Study of Health in Pomerania (SHIP) cohort study, a decline in TPOAb positivity was reported (from 3.9% to 2.9%) [31]. However, this may be due to the high positivity cut-off (>200 IU/mL), potentially underestimating the true prevalence of TPOAb positivity.

Regarding the incidence of TPOAb positivity, our findings align with our earlier study within the framework of TTS published by Amouzegar et al. that reported an incidence of 7.12 per 1,000 person-years up to 2012, with higher rates in women than in men [12]. The Brazilian Longitudinal Study of Adult Health (ELSA-Brasil) study found the 4-year cumulative incidence of TPOAb positivity to be 1.46% in the total population, 1.23% in men, and 1.67% in women [14]. Li et al. reported a 5-year cumulative incidence of 2.81%, with no sex-based differences [15]. The differences in the reported rates could be due to the interplay between genetic factors and environmental exposures in different regions [17,18]. Additionally, the absence of the same reference standards for TPOAb cut-off determination and antibody detection assays plays an essential role in these differences [32].

The observed increasing trend of TPOAb positivity may reflect the influence of multiple factors, including environmental pollution (for example, air pollution and endocrine-disrupting chemicals), urbanization (such as increased population density and changes in residential environments), lifestyle changes (including shifts in dietary patterns and reduced physical activity), and nutritional status, particularly iodine intake [[33], [34], [35]]. In addition to these factors, genetic susceptibility may contribute to the rising frequency of TPOAb. In a longitudinal analysis from the TTS, several variants within the TPO gene were found to be associated with both prevalent TPOAb positivity and incident TPOAb seroconversion, independent of age, sex, BMI, smoking status, parity, and oral contraceptive use, with rs6605278 showing the strongest and most consistent association [18]. These findings suggest that genetic predisposition may interact with environmental and nutritional exposures to influence TPOAb development. Several studies have indicated that mild-to-moderate iodine deficiency can trigger AITD [35]. Although Iran has been classified as an iodine-sufficient country based on several national monitoring surveys [[21], [22], [23], [24]], these data indicate a downward trend in median urinary iodine concentrations (UIC) among school-aged children in recent years. Therefore, the decreasing trend of sufficient iodine levels in Iran, coupled with inadequate intake among vulnerable populations, especially in pregnant women, suggests a potential emergence of iodine deficiency that may increase the risk of AITD.

As expected, female sex was a strong risk factor for TPOAb positivity. This confirms the results of previous studies that have explored this association [4,5,12]. Other nonthyroidal autoantibodies are also more common in women than in men [2]. TSH ≥5 mU/L is strongly associated with TPOAb positivity, a relationship supported by several studies [12,36]. Age <40 years was positively associated with incident TPOAb positivity, indicating that younger age may increase susceptibility to AITD. This finding is consistent with several large population-based studies [5,12]. In univariate analysis, current smoking and parity were significantly associated with TPOAb positivity; however, these associations were no longer significant in multivariate analysis. This finding regarding parity aligns with evidence from large-scale longitudinal studies, which initially observed positive associations between parity and AITD but found no significant relationship after adjusting for age, smoking, and other confounders [37,38].

The present study has several important strengths. This study represents the longest population-based cohort worldwide, focused on TPOAb over the past two decades across five repeated measurements. In contrast to studies relying on secondary data from electronic health records (EHR), this study was specifically designed to investigate TPOAb using standardized laboratory protocols and identical assay kits throughout all measurements, ensuring consistency and reliability. To our knowledge, this is the first study that evaluates the long-term trend of TPOAb positivity and its projection for 2030. Some limitations should also be considered. The findings may not be fully generalizable to other populations due to differences in ethnicity, geographic region, and iodine sufficiency. Because no new participants were enrolled at follow-up, the same cohort was observed over time, which results in cohort aging during the 18-year follow-up. Although age- and sex-standardization is applied to reduce this bias, some residual effects may remain. Finally, the study did not collect data on the family history of AITD, which is known to influence individuals’ TPOAb positivity.

These findings underscore the need for targeted monitoring strategies and public health measures to ensure adequate iodine nutrition in high-risk groups, particularly women of childbearing age, as recent evidence has suggested TPOAb positivity as the single most significant risk factor for the development of thyroid dysfunction during pregnancy [39]. Future research should investigate environmental, genetic, and dietary factors contributing to the rising trend of TPOAb positivity.

In conclusion, this is the first and longest study worldwide that demonstrated an upward trend in TPOAb positivity over 18 years across five repeated measurements in an iodine-sufficient population, driven by female sex, age <40, and TSH ≥5 mU/L. TPOAb positivity in this cohort is projected to reach 21.04% in 2030. Trajectory patterns of TPOAb showed that the majority of participants had consistently low-stable levels of TPOAb, and smaller subgroups followed low-increasing, high-decreasing, or high-stable trends over time.

## CRediT authorship contribution statement

**Mohamadamin Tarighat-Payma:** Writing – review & editing, Writing – original draft, Visualization, Methodology, Investigation, Formal analysis, Conceptualization. **Ladan Mehran:** Writing – review & editing, Methodology, Conceptualization. **Safdar Masoumi:** Writing – review & editing, Visualization, Methodology, Formal analysis, Data curation. **Maryam Tohidi:** Writing – review & editing, Resources, Methodology. **Atieh Amouzegar:** Writing – review & editing, Validation, Supervision, Methodology, Conceptualization. **Fereidoun Azizi:** Writing – review & editing, Validation, Resources, Project administration. **Elizabeth N. Pearce:** Writing – review & editing, Validation, Supervision.

## Availability of data and materials

Datasets generated during and analyzed during the current study are not publicly available due to institutional policies, but are available from the corresponding author upon reasonable request.

## Ethical approval

This study conformed to the ethical guidelines of the Helsinki Declaration and was approved by the ethics committee of Research Institute for Endocrine Sciences (RIES) of Shahid Beheshti University of Medical Sciences (code: IR.SBMU.ENDOCRINE.REC.1404.030). All participants provided informed written consents.

## Funding

This study was supported in part by grant No. 43013419-6 from 10.13039/501100005851Shahid Beheshti University of Medical Sciences.

## Declaration of competing interest

The authors declare that they have no known competing financial interests or personal relationships that could have appeared to influence the work reported in this paper.

## Data Availability

Data will be made available on request.
