# Supplementary material for: Long-term natural history of thyroid peroxidase antibodies in a population-based cohort: Findings from 18 years of follow-up in Tehran Thyroid Study (TTS)
Source: J Transl Autoimmun. 2026 Feb 16;12:100358. doi: 10.1016/j.jtauto.2026.100358 (PMC12933471; doi:10.1016/j.jtauto.2026.100358)
Supplement: Multimedia component 1 [file mmc1.docx]

**Supplementary Table 1.** Baseline characteristics of the study populations stratified by trajectory classes.

|  | Class 1  (n=723) | Class 2  (n=129) | Class 3  (n=4,426) | Class 4  (n=160) | P value |
| --- | --- | --- | --- | --- | --- |
| Sex, n (%) |  |  |  |  | <0.001 |
| Male | 201 (27.8) | 37 (28.7) | 2019 (45.6) | 48 (30.0) |  |
| Female | 522 (72.2) | 92 (71.3) | 2407 (54.4) | 112 (70.0) |  |
| Age (years) | 41.7 ± 41.0 | 44.9 ± 44.0 | 40.6 ± 39.0 | 30.1 ± 31.0 | <0.001 |
| BMI (kg/m2) | 27.5 ± 4.9 | 27.8 ± 3.9 | 26.6 ± 4.5 | 25.2 ± 4.3 | <0.001 |
| Waist circumference (cm) | 88.2 ± 12.6 | 89.9 ± 10.1 | 87.2 ± 12.0 | 81.4 ± 10.2 | <0.001 |
| Education level |  |  |  |  | 0.008 |
| Primary school | 314 (43.4) | 67 (51.9) | 1704 (38.5) | 36 (22.5) |  |
| High school | 309 (42.7) | 50 (38.8) | 2103 (47.5) | 99 (61.9) |  |
| Higher education | 100 (13.8) | 12 (9.3) | 619 (14.0) | 25 (15.6) |  |
| Smoking status |  |  |  |  | 0.038 |
| Non-smoker | 651 (90.0) | 115 (89.1) | 3908 (88.3) | 144 (90.0) |  |
| Smoker | 72 (10.0) | 14 (10.9) | 518 (11.7) | 16 (10.0) |  |
| Low physical activity | 250 (34.6) | 37 (28.6) | 1691 (38.2) | 56 (35.6) | 0.8 |
| TSH (mU/L) | 5.7 ± 14.8 | 9.7 ± 34.8 | 2.0 ± 7.6 | 2.7 ± 4.6 | <0.001 |
| FT4 (ng/dl) | 1.2 ± 1.1 | 1.2 ± 0.5 | 1.2 ± 0.2 | 1.2 ± 0.2 | <0.001 |

Categorical variables were reported as count (percentage), and continuous variables as mean ± SD or median (IQR). Abbreviations: BMI, body mass index; TSH, thyroid-stimulating hormone; FT4, free T4; TPOAb, thyroid peroxidase antibody.

# Supplementary Table 2. Observed (2018), model-estimated (2018), and model-projected (2030) prevalence of TPOAb positivity

| **Year** | **Data Type** | **Prevalence of TPOAb positivity (95% CI)** |
| --- | --- | --- |
| 2018 | Observed | 16.35% (14.6-18.3) |
| 2018 | Model Estimate | 16.7% (15.6-17.9) |
| 2030 | Model Projection | 21.04% (16.01-26.08) |

Abbreviations: TPOAb, thyroid peroxidase antibody.

# Supplementary Table 3. Distribution of participants based on the number of visits with positive TPOAb results (out of five repeated visits)

| **Count of measurements with TPOAb positivity per participant** | **Frequency (%)** |
| --- | --- |
| Negative in all visits | 4330 (79.62) |
| Positive in 1 visit | 226 (4.16) |
| Positive in 2 visits | 174 (3.20) |
| Positive in 3 visits | 210 (3.86) |
| Positive in 4 visits | 278 (5.11) |
| Positive in all 5 visits | 220 (4.05) |

Abbreviations: TPOAb, thyroid peroxidase antibody.

| **Latent Class** | **Proportion of participants (%)** | **TPOAb**  **(Logarithmic scale)** |
| --- | --- | --- |
| Class 1 | 13.3 | 5.1 to 5.7 |
| Class 2 | 2.4 | 7.8 to 0.8 |
| Class 3 | 81.4 | 1.8 to 1.9 |
| Class 4 | 2.9 | 0.9 to 9.7 |

# Supplementary Table 4. Distribution and characteristics of latent classes of TPOAb trajectories over 18 years of follow-up

Abbreviations: TPOAb, thyroid peroxidase antibody.

|  | 1999-2002  (First visit) | 2002-2005  (Second visit) | 2005-2008  (Third visit) | 2008-2011  (Fourth visit) | 2015-2018  (Fifth visit) | P value for trend |
| --- | --- | --- | --- | --- | --- | --- |
| TSH (mU/L) |  |  |  |  |  |  |
| Class 1 | 5.7 ± 14.8 | 7.0 ±23.2 | 7.0 ± 16.4 | 7.6 ± 29.7 | 6.9 ± 18.7 | 0.37 |
| Class 2 | 9.7 ± 34.8 | 6.3 ±16.6 | 6.4 ± 22.5 | 5.3 ± 17.0 | 4.2 ± 9.8 | 0.002 |
| Class 3 | 2.0 ± 7.6 | 2.2 ±7.5 | 2.2 ± 3.8 | 2.5 ± 4.8 | 2.5 ± 2.6 | <0.001 |
| Class 4 | 2.7 ± 4.6 | 2.8 ±2.4 | 3.9 ± 5.9 | 3.9 ± 3.8 | 5.7 ± 9.2 | <0.001 |
| P value | <0.001 | <0.001 | <0.001 | <0.001 | <0.001 |  |
| FT4 (ng/dl) |  |  |  |  |  |  |
| Class 1 | 1.2 ± 1.1 | 1.2 ± 0.3 | 1.1 ± 0.3 | 1.1 ± 0.3 | 1.2 ± 0.4 | 0.59 |
| Class 2 | 1.2 ± 0.5 | 1.1 ±0.3 | 1.1 ± 0.3 | 1.2 ± 0.3 | 1.2 ± 0.3 | 0.08 |
| Class 3 | 1.2 ± 0.2 | 1.2 ± 0.3 | 1.2 ± 0.2 | 1.2 ± 0.2 | 1.2 ± 0.2 | 0.06 |
| Class 4 | 1.2 ± 0.2 | 1.2 ± 0.39 | 1.2 ± 0.2 | 1.2 ± 0.2 | 1.2 ± 0.6 | 0.72 |
| P value | 0.34 | 0.34 | 0.35 | 0.35 | 0.34 |  |
| BMI (kg/m2) |  |  |  |  |  |  |
| Class 1 | 27.5 ± 4.9 | 28.2 ± 5.0 | 27.2 ± 4.7 | 28.1 ± 4.0 | 29.3 ± 5.3 | <0.001 |
| Class 2 | 27.8 ± 3.9 | 28.6 ± 4.0 | 28.3 ± 4.5 | 28.4 ± 4.5 | 28.5 ± 4.2 | <0.001 |
| Class 3 | 26.6 ± 4.5 | 27.3 ± 4.4 | 27.8 ± 4.3 | 28.0 ± 4.7 | 28.6 ± 4.7 | <0.001 |
| Class 4 | 25.2 ± 4.3 | 26.6 ± 4.7 | 27.0 ± 4.1 | 27.9 ± 4.4 | 28.9 ± 4.8 | <0.001 |
| P value | <0.001 | <0.001 | 0.02 | 0.08 | 0.12 |  |

**Supplementary Table 5.** Longitudinal changes in TSH, FT4, and BMI across study phases by trajectory class

Abbreviations: Body mass index; BMI; TSH, thyroid-stimulating hormone; FT4; Free Thyroxine

# Supplementary Table 6. Age- and sex-standardized prevalence of TPOAb positivity in 2018 based on age and sex

|  | TPOAb Positivity Status | | TPOAb Ranges (IU/mL) | | | | |
| --- | --- | --- | --- | --- | --- | --- | --- |
|  | Total population | Persistent group | <5 | 5-15 | 15-35 | 35-500 | ≥500 |
|  | Prevalence rate  (95 % CI) | Prevalence rate  (95 % CI) | Prevalence rate  (95 % CI) | Prevalence rate  (95 % CI) | Prevalence rate  (95 % CI) | Prevalence rate  (95 % CI) | Prevalence rate  (95 % CI) |
| Total | 16.3  (14.6-18.3) | 8.5  (7.3-9.9) | 47.1 (44.6-49.6) | 30.1 (27.8-32.4) | 6.5 (5.4-7.8) | 13.7 (12.1-15.5) | 2.7 (2.0-3.6) |
| < 40 years | 15.7  (13.1-18.7) | 7.3  (5.6-9.4) | 47.4  (43.6-51.2) | 30.6  (27.2-34.2) | 6.3  (4.7-8.5) | 13.1  (10.8-15.9) | 2.6  (1.6-4.1) |
| 40-60 years | 18.8  (17.1-20.6) | 10.2 (9.0-11.6) | 45.4  (43.2-47.6) | 29.1  (27.1-31.1) | 6.8  (5.7-8.0) | 15.6  (14.1-17.3) | 3.2  (2.5-4.1) |
| ≥ 60 years | 14.4  (12.7-16.4) | 11.9 (10.-13.6) | 49.4  (46.8-52.0) | 29.2  (26.9-31.6) | 7.0  (5.8-8.4) | 12.4  (10.8-14.2) | 2.0  (1.4-2.9) |
| Men | 11.2  (8.9-13.9) | 4.8  (3.6-6.6) | 53.8 (49.7-57.9) | 30.3 (26.6-34.2) | 4.7 (3.3-6.6) | 8.6 (6.7-11.1) | 2.6 (1.5-4.3) |
| < 40 years | 10.4  (7.1-14.8) | 3.9  (2.2-6.9) | 55.0  (48.8-61.0) | 30.7  (25.3-36.7) | 4.0  (2.2-7.3) | 7.6  (4.9-11.6) | 2.8  (1.3 -5.7) |
| 40-60 years | 13.9  (11.7-16.5) | 6.2  (4.8-8.0) | 52.0  (48.5-55.5) | 28.2  (25.2-31.5) | 5.9  (4.4-7.8) | 11.2  (9.2-13.7) | 2.7  (1.8-4.1) |
| ≥ 60 years | 9.6  (7.4-12.2) | 7.6 (5.7-10.0) | 51.2  (47.2-55.2) | 32.9  (29.2-36.8) | 6.4  (4.7-8.6) | 8.6  (6.6-11.1) | 1.0  (0.5-2.2) |
| Woman | 19.6  (17.2-22.2) | 10.9  (9.2-12.9) | 42.9 (39.8-46.0) | 29.9 (27.1-32.8) | 7.6 (6.1-9.5) | 16.9 (14.6-19.3) | 2.7 (1.-3.9) |
| < 40 years | 18.9  (15.4-23.0) | 9.5 (7.1-12.6) | 42.7  (38.0-47.6) | 30.6  (26.3-35.2) | 7.8  (5.5-10.8) | 16.5  (13.2-20.4) | 2.4  (1.3-4.5) |
| 40-60 years | 22.0  (19.7-24.4) | 13.0 (11.2-14.9) | 41.1  (38.3-43.9) | 29.6  (27.1-32.3) | 7.4  (6.0-9.0) | 18.5  (16.4-20.8) | 3.5  (2.6-4.7) |
| ≥ 60 years | 17.9  (15.5-20.7) | 14.9 (12.7-17.5) | 48.1  (44.7-51.5) | 26.5  (23.6-29.7) | 7.4  (5.8-9.4) | 15.2  (12.9-17.8) | 2.8  (1.9-4.2) |

Abbreviations: TPOAb, thyroid peroxidase antibody.

|  | TPOAb Positivity Status | | TPOAb Ranges (IU/mL) | | | | |
| --- | --- | --- | --- | --- | --- | --- | --- |
|  | Total population | Persistent group | <5 | 5-15 | 15-35 | 35-500 | ≥500 |
|  | Prevalence rate  (95 % CI) | Prevalence rate  (95 % CI) | Prevalence rate  (95 % CI) | Prevalence rate  (95 % CI) | Prevalence rate  (95 % CI) | Prevalence rate  (95 % CI) | Prevalence rate  (95 % CI) |
| Smoking status | | | | | | | |
| Non-smoker | 16.5  (14.6-18.5) | 8.5 (7.3-10.0) | 45.6  (43.0 -48.3) | 30.9  (28.5-33.4) | 6.9  (5.7-8.4) | 13.9  (12.2 ,15.9) | 2.5  (1.8-3.5) |
| Smoker | 15.3  (10.6-21.6) | 7.2 (2.6-18.6) | 59.2  (51.5-66.4) | 22.7  (17.0-29.7) | 2.8  (1.4-5.6) | 11.4  (7.4-17.2) | 3.8  (1.7-8.3) |
| BMI | | | | | | | |
| Normal weight | 16.3  (13.1-20.2) | 7.9 (5.9-10.6) | 47.1  (42.4-51.8) | 28.2  (24.1-32.6) | 8.4  (6.0-11.6) | 14.5  (11.4-18.3) | 1.8  (1.0-3.3) |
| Overweight | 16.0  (13.4-19.0) | 7.6 (5.9-9.7) | 47.0  (43.2-50.9) | 30.9  (27.4-34.6) | 6.1  (4.6-8.1) | 12.9  (10.6-15.7) | 3.1  (1.9-4.8) |
| Obese | 16.1  (13.0-19.7) | 10.1 (7.8-13.0) | 47.8  (43.0-52.7) | 30.6  (26.3-35.3) | 5.5  (3.7-8.1) | 13.0  (10.2-16.3) | 3.1  (1.8-5.2) |
| TSH (mU/L) | | | | | | | |
| < 0.3 | 26.0  (14.0-43.1) | 11.2 (2.8-35.6) | 39.0  (24.3-56.0) | 28.2  (15.9-45.0) | 6.9  (1.9-21.5) | 21.5  (11.0-37.8) | 4.5  (0.7-23.7) |
| 0.3 -2 | 8.8  (6.8-11.3) | 5.9 (3.8-9.0) | 55.6  (51.6-59.4) | 31.4  (27.9-35.2) | 4.2  (3.0-5.9) | 7.9  (6.1-10.3) | 0.8  (0.4-1.9) |
| 2 - 5 | 15.0  (12.6-17.8) | 9.6 (6.8-13.5) | 45.0  (41.4-48.8) | 32.3  (28.9-35.9) | 7.6  (5.8-9.9) | 13.8  (11.5-16.5) | 1.2  (0.7-2.3) |
| ≥ 5 | 46.9  (39.6-54.4) | 38.8 (27.4-51.6) | 26.4  (20.3-33.5) | 16.2  (11.6-22.3) | 10.4  (6.6-16.1) | 32.5  (26.0-39.9) | 14.4  (9.8-20.6) |

# Supplementary Table 7. Age- and sex-standardized prevalence of TPOAb positivity in 2018 based on smoking, BMI categories and TSH categories

Abbreviations: Body mass index; BMI; TSH, thyroid-stimulating hormone.


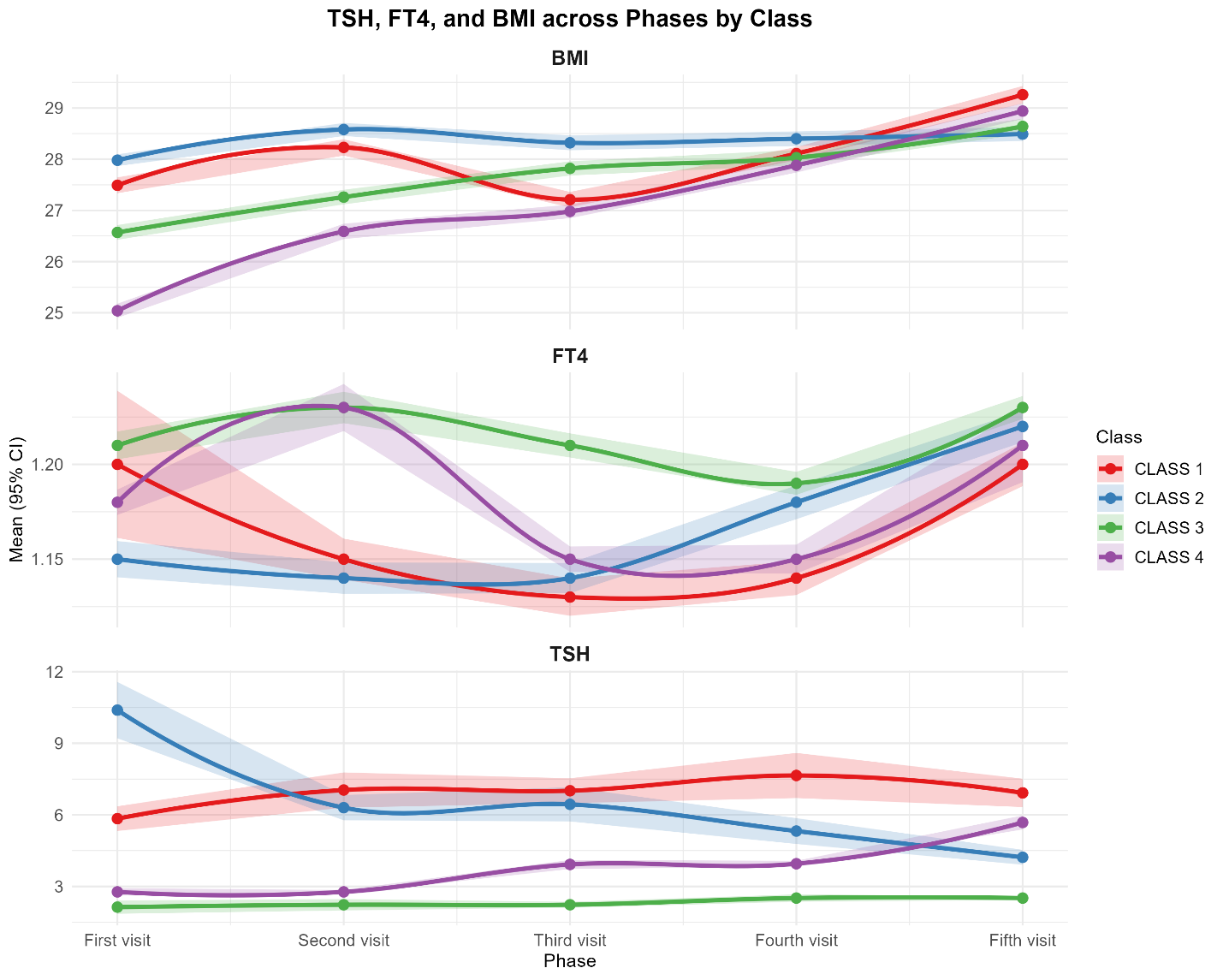


**Supplementary Figure 1.** Longitudinal trends of body mass index (BMI), free thyroxine (FT4), and thyroid-stimulating hormone (TSH) across study phases stratified by latent classes. Mean values with 95% confidence intervals are shown for each class at each visit. Class 1 is shown in red, Class 2 in blue, Class 3 in green, and Class 4 in purple. The upper panel depicts BMI (kg/m²), the middle panel FT4 (ng/dL), and the lower panel TSH (mU/L) from the first visit (1999-2002) to the fifth visit (2015-2018).
